# *In vitro* chondroprotective potential of *Senna alata* and *Senna tora* in porcine cartilage explants and their species differentiation by DNA barcoding-high resolution melting (Bar-HRM) analysis

**DOI:** 10.1371/journal.pone.0215664

**Published:** 2019-04-19

**Authors:** Siriwan Ongchai, Chatchadawalai Chokchaitaweesuk, Patiwat Kongdang, Siriwadee Chomdej, Kittisak Buddhachat

**Affiliations:** 1 Thailand Excellence Center for Tissue Engineering and Stem Cells, Department of Biochemistry, Faculty of Medicine, Chiang Mai University, Chiang Mai, Thailand; 2 Department of Biology, Faculty of Science, Chiang Mai University, Chiang Mai, Thailand; 3 Excellent Center in Veterinary Bioscience, Chiang Mai University, Chiang Mai, Thailand; Fu Jen Catholic University, TAIWAN

## Abstract

*Senna* species and anthraquinone derivatives generated by these organisms, rhein and aloe-emodin, exert anti-inflammatory effects. These species present a similar morphology but produce different ingredients when they are used as medicinal products. In this study, a DNA barcoding- (Bar-) high-resolution melting (HRM) technique was developed using internal transcribed sequence 2 (ITS2) to differentiate between *Senna alata* and *Senna tora* as a result of significant differences in their melting profiles. We used this approach for confirmation of *S*. *alata* and *S*. *tora* raw materials, and we examined the chondroprotective properties of the ethanolic extracts of *S*. *alata* and *S*. *tora* using a porcine model of cartilage degradation induced by a combination of interleukin-17A (IL-17A) and IL-1β. We found that both *Senna* ethanolic extracts, at a concentration of 25 μg/mL, effectively prevented cartilage degradation. Rhein and aloe-emodin were present in the extract of *S*. *alata* but not in that of *S*. *tora*. We observed a reduction in the release of sulfated glycosaminoglycans (S-GAGs) and hyaluronic acid (HA) into media in both treatments of *Senna* extracts, which indicated proteoglycan preservation in explant tissues. These results suggest that neither rhein nor aloe-emodin are the main factors responsible for cartilage-protecting properties. Taken together, results show that both *S*. *alata* and *S*. *tora* are promising for further development as anti-osteoarthritic agents and that Bar-HRM using ITS2 could be applied for species confirmation with *Senna* products.

## Introduction

Osteoarthritis (OA) involves joint degeneration, especially in the elderly, and is caused by proinflammatory cytokines (e.g., interleukin-1 (IL-1), tumor necrosis factor α (TNFα), oncostatin M (OSM), IL-6, and IL-17), leading to an imbalance in the biochemical processes in articular cartilage [1–3]. Cytokines IL-1β and IL-17A are detected in synovial fluid (SF) of rheumatoid arthritis (RA) and OA patients [3–5]. These compounds are well known to be involved in the inflammatory process and cartilage degradation, by which IL-17A strongly synergizes with other inflammatory cytokines (e.g., IL-1, TNFα, IL-6) to stimulate cartilage collagen and proteoglycan breakdown via matrix-degrading enzymes (e.g., matrix metalloproteinase (MMP) 1, 3, and 13) [1–3, 6]. Current OA therapy is only moderately effective as a treatment option, especially with pain relieving drugs (e.g., non-steroid anti-inflammation drugs with adverse effects) [7, 8]. Presently, disease-modifying osteoarthritis drugs (DMOAD) have been extensively studied for the ability to halt the structural disease progression of OA and also to improve symptoms and/or function [9]. Naturally-derived or occurring ingredients from medicinal plants are a promising source for drug discovery for DMOAD.

The *Senna* genus is comprised of approximately 260–350 species, including herbs, shrubs, and trees, belonging to the legume family Fabaceae and the subfamily Caesalpinioideae [10, 11]. *Senna alata* (L.) Roxb. and *Senna tora* (L.) Roxb. are frequently used for medicinal purposes, including as components of laxatives [12, 13], anti-inflammatory agents [14, 15], anti-cancer drugs [15], and hepatoprotectors [16]. Anthraquinones (e.g., rhein (1,8-dihydroxyanthraquinone-3-carboxylic acid) and aloe-emodin (1,8-dihydroxy-3-(hydroxymethyl)-anthraquinone)) are the main bioactive ingredients of *Senna* species and exhibit antioxidant and anti-inflammatory activities [17, 18]. Rhein is an anthraquinone derivative that is present in *Rheum* species and in the leaves of several *Senna* species and has previously been reported to decrease the production of interleukin-1β (IL-1β) and matrix metalloproteinases in articular chondrocytes [19–21]. Aloe-emodin is an anthraquinone found in the leaves of *Senna* plants that has various properties, including anti-cancer activity [22], hepatoprotection against carcinogen-induced liver damage [23], and anti-inflammatory effects [24].

Both *S*. *alata* and *S*. *tora* present a similar morphology and have a similar Thai common name (Chumhet-tet and Chumhet-thai, respectively). However, their anthraquinones are significantly different in several *Senna* species [17], which are likely to influence their biological activities. In addition, the raw materials produced by these plants are usually processed and/or modified into various products, such as powder capsules and herbal infusions, which can further contribute to difficulty in the species differentiation [25–28]. Accurate species identification during the manufacturing process and data generation to support the use of herbal plants as complementary and alternative medicine (CAM) options is necessary for the safety, quality, and efficacy of medicinal plant ingredients [25–28]. A DNA barcoding- (Bar-) high-resolution melting (HRM) method, “Bar-HRM,” was employed here to differentiate *S*. *alata* and *S*. *tora* given that we expect the sequence of internal transcribed sequence 2 (ITS2) of each species to be different [28, 29]. Thereby, when disassociated from double strand to single strand, DNA from the different species will give a different melting temperature that is a unique characteristic to that organism dependent on base sequence, length, and neighboring DNA [30, 31]. In addition, Bar-HRM can used for detecting adulteration. When herbal products admixed with more than one species, the melting profile obtained via Bar-HRM will present more than one melting peak that is derived from the admixture of DNA products from other species [25, 27, 32].

The aim of this study was to establish a novel and rapid method to distinguish between *S*. *alata* and *S*. *tora* using Bar-HRM with internal transcribed sequence 2 (ITS2) and to evaluate the chondroprotective potential of *S*. *alata* and *S*. *tora* extracts, as well as rhein and aloe-emodin, using a cartilage explant model.

## Materials and methods

### Samples collection and preparation

*S*. *alata* and *S*. *tora* dried leaves were bought from Lampang Herb Conservation, Lampang, Thailand (www.lampangherbs.com), and identified by the taxonomist Wannaree Charoensup. A voucher specimen is stored under the numbers 023194 (*S*. *alata*) and 023193 (*S*. *tora*) at the medicinal plant herbarium, Faculty of Pharmacy, Chiang Mai University, Thailand. Fresh leaves were used for species authentication by Bar-HRM, and dried leaves were used for the phytochemical analysis and chondroprotective assay.

Dried leaves were blended into fine powder using herb grinder machine and then macerated in 80% ethanol (1 g powder per 10 mL ethanol) for three days at room temperature. The extracts were evaporated at 45°C under a 150 mbar pressure using a rotary evaporator (Buchi Rotavapor R-200, Switzerland)). Crude extracts were stored at −20°C for further use.

### Distinguishing the plants species using HRM analysis plant materials and DNA isolation

The fresh leaves of *S*. *alata* and *S*. *tora* (100 mg) were ground under liquid nitrogen prior to DNA isolation using NucleoSpin Plant II (Macherey-Nagel) according to the manufacturer’s instructions. The DNA concentration was determined using a NanoDrop 2000/2000c spectrophotometer (ThermoFisher Scientific, USA) and adjusted to 20 ng/μL.

#### Primer design and HRM analysis

ITS2 sequences of *S*. *alata*, *S*. *tora*, and other closely related species were retrieved from GenBank (*S*. *alata*: HQ833041, FJ980412, and JQ301818; *S*. *tora*: FJ572046, KJ638424, and JQ301837; *S*. *siamea*: JQ301842; *S*. *spectabilis*: JQ301836; *S*. *alexandrina*: JQ301846; *S*. *hirsuta*: JQ301839; and *S*. *occidentalis*: JQ301840). The criteria for HRM primer design were as follows: (i) the primer binding sites were the conserved regions for the species/groups to amplify, (ii) there were variable nucleotides within the amplicon of the different species/groups, and (iii) the amplicon size did not exceed 300 base pairs. ITS2-derived *Senna*-specific primers were tested for species differentiability using HRM.

HRM was employed using 7500/7500 Fast Real-Time PCR System (Applied Biosystems, USA). KAPA Taq PCR Kit (Kapa Biosystems, USA) was used for the DNA amplification step. The forward primer was 5′-TCTGCCTGGGTGTCACGCATCGTTG-3′ and the reverse primer was 5′-GGTAGCCCCGCCTGACCTGGGGTC-3′. An initial denaturing step was performed at 95°C for 5 min, followed by 35 cycles at 95°C for 30 s, 60°C for 30 s, and 72°C for 20 s. The fluorescence value was recorded at the end of each extension step. For HRM, the products were denatured at 95°C for 15 s and then annealed at 60°C for 15 s. Fluorescence data were collected at every 0.1°C to reach 95°C and transformed into a normalized plot and different plot for better visualization in species discrimination. The melting temperature (T_m_) was analyzed using the HRM, and the HRM products were subjected to 1.5% agarose gel electrophoresis at 100 V for 30 min in 1x Tris-borate-EDTA buffer and then stained with ethidium bromide to visualize DNA under UV illuminator. PCR products obtained from *S*. *alata* and *S*. *tora* were sequenced to confirm the specificity of HRM results and the sequences were deposited in GenBank accession number MK685898 and MK685899, respectively. In addition, we tested the ability of Bar-HRM using ITS2 for detecting admixture between *S*. *alata* and *S*. *tora* by mixing DNA of *S*. *alata* (20 ng/μL) and *S*. *tora* (20 ng/μL) at different proportions, given as 10, 25, 50, 75, and 90% of *S*. *alata* in *S*. *tora*.

### Phytochemical analysis

#### Determination of total anthraquinones

Crude extracts and aloe-emodin (Selleck Chemicals; purity = 99.09%) were dissolved in 0.5% (w/v) magnesium acetate in methanol. The total level of anthraquinones of each extract was determined as previously described [33] and calculated using the aloe-emodin standard curve (0–10 μg/mL).

#### Determination of rhein and aloe-emodin using high-performance liquid chromatography (HPLC)

Crude extracts, aloe-emodin, and rhein (Sigma-Aldrich; purity > 98%) were dissolved in dimethyl sulfoxide (DMSO). The phytochemical profile of each extract was measured by the HPLC technique. The mobile phases were a mixture of 0.05 M ammonium acetate and acetonitrile under the following gradient: 0–10 min, 70%:30%; 10–14 min, 20%:80%; and 14–15 min, 70%:30%, respectively. The extracts were then separated on a Hypersil C18 column (250 × 4.6 mm, 5 μm; Agilent) as previously described [34]. Commercial compounds were used as standards at concentrations ranging from 0 to 20 μg/mL.

#### Determination of total phenolic compounds

The total phenolic compounds were measured from the extracts (500 μg/mL dissolved in absolute methanol) using the Folin–Ciocalteu method [35]. The amount of total phenolic compounds was displayed in gallic acid equivalent, which was calculated from the gallic acid standard curve (0–50 μg/mL).

### Determination of the antioxidant activity

The 1,1-diphenyl-2-picrylhydrazyl (DPPH) radical scavenging activity was measured using a modified method [36]. Briefly, 50 μL of each extract concentration (dissolved in methanol) was mixed with 150 μL of 0.1 mM DPPH solution and then incubated at room temperature for 30 min and protected from light. The reaction was read at 520 nm. Ascorbic acid was used as the positive control.

### Evaluation of the chondroprotective potential of *S*. *alata* and *S*. *tora* using a cartilage explant model

#### Cartilage explant preparation and treatments

**Fig 1:**
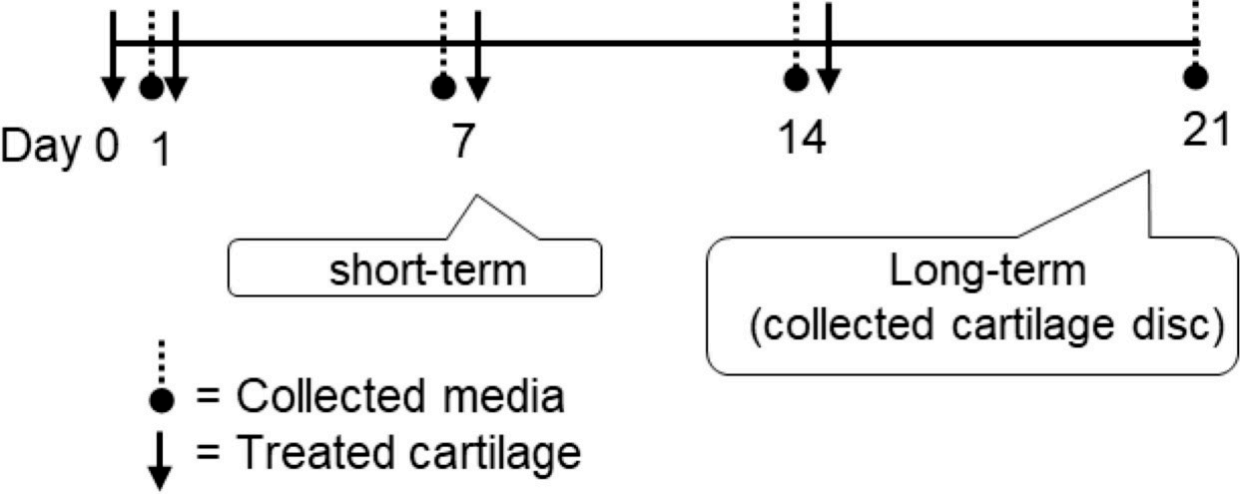
Timeline of treatments and sample collection for the assessment of chondroprotection in a cartilage explant culture model.

Porcine articular cartilages were prepared from the metacarpophalangeal joints (from a local slaughterhouse) as previously described [37]. Briefly, the cartilage explants were cultured in serum-free DMEM (Gibco) containing 10% penicillin-streptomycin in a humidified incubator at 37°C for 24 h. The cartilage tissues were then pretreated with the combined cytokines, 2 ng/mL IL-1β and 4ng/mL IL-17A, for 2 h prior to cotreatment with crude extracts (12.5–50 μg/mL) and active compounds (rhein or aloe-emodin) at 1.25, 2.5, and 5 μg/mL. The diacerein, anti-osteoarthritic agent, at 50 μM was served as positive control. For short-term culture, the culture media were harvested on day 7 to measure the release of S-GAGs and HA. For long-term culture, cytokines-pretreated cartilage tissues were treated with either 25 μg/mL crude extracts or 5 μg/mL active compounds to evaluate the cumulative releasing profile of S-GAGs and HA in the culture media on days 0, 7, 14, and 21 (Fig 1).

#### Evaluation of cartilage degradation

The release of S-GAGs and HA in the culture medium was determined by a colorimetric dye binding assay and ELISA technique, respectively, as previously described [38, 39].

#### Histological examination

On day 21, the cartilage tissues fixed in 4% paraformaldehyde were embedded in paraffin blocks and cut into 5 μm thick samples. H&E staining was used for the morphological observation of chondrocytes. Safranin O staining was used to detect proteoglycan accumulation [40].

#### Evaluation of cell viability by LDH and MTT assays

Following treatment with the test agents, the cartilage explant model was assessed for cell viability by measuring the release levels of LDH in the media, while the MTT assay was employed for cell cytotoxicity evaluation after human articular chondrocytes were exposed to the test agents as previously described [37].

### Statistical analysis

The difference between either control and cytokines-induced group or cytokines-induced group and treatments was performed by a Student’s T-test. Results are expressed as mean ± standard deviation (SD) from triplicate samples of three independent experiments. All data in this study was recoded in S1 File.

## Results

### Bar-HRM for species differentiation

**Fig 2:**
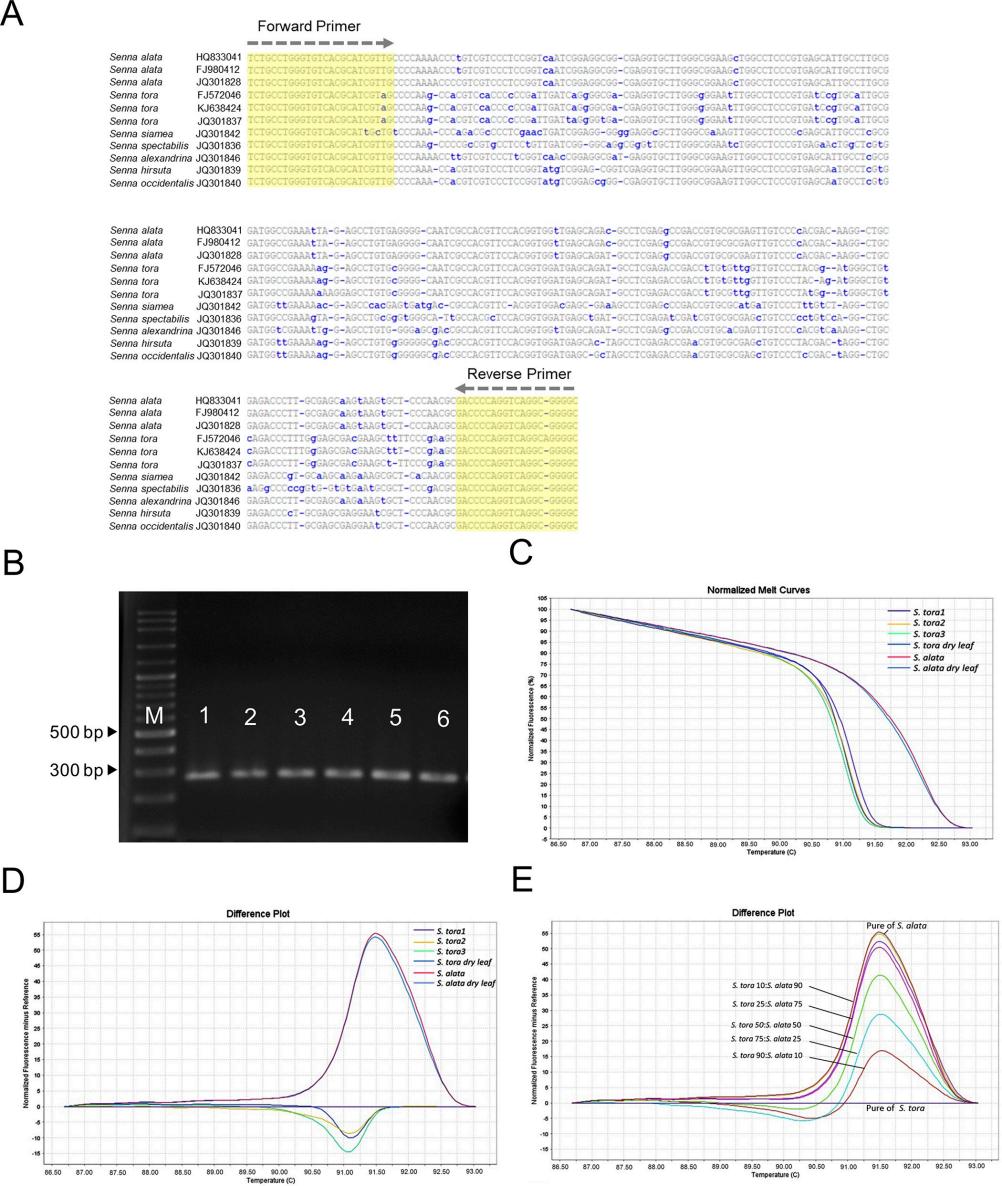
Distinction between *S*. *tora* and *S*. *alata* on the basis of DNA Bar-HRM using ITS2. (A) Multiple alignment of *S*. *alata* and *S*. *tora*. (B) Agarose gel electrophoresis of a 280 bp amplicon of an ITS2 locus amplified from *S*. *alata* and *S*. *tora*. Lanes 1–3: *S*. *tora* (fresh leaves); Lane 4: *S*. *tora* (dried leaves); Lane 5: *S*. *alata* (fresh leaves); Lane 6: *S*. *alata* (dried leaves). The DNA ladder size is indicated in base pairs. (C) The normalized melt curves profiles of *S*. *tora* and *S*. *alata* species (both in fresh leaves and in dried leaves) using Bar-HRM using ITS2. (D) The difference plots of Bar-HRM using ITS2 of *S*. *alata*. *S*. *tora* were performed as a reference genotype (purple baseline). (E) The difference plot for adulteration detection of *S*. *tora* at 10, 25, 50, 75, and 90% in *S*. *alata*. The baseline (purple) and top line represent the unadulterated sample of *S*. *tora* and *S*. *alata*, respectively.

In the present study, Bar-HRM was established using the ITS2 region in order to differentiate between the species *S*. *alata* and *S*. *tora*, which are closely related. A primer pair was designed on the ITS2 region, in which the amplified sequences displayed the variation between them, as shown in Fig 2A. The primer pair yielded a 280 bp amplicon in either species (Fig 2B). The polymerase chain reaction (PCR) products were then confirmed by DNA sequencing as similar PCR sequences were obtained for both *S*. *alata* (MK685898) and *S*. *tora* (MK685899). The ITS2 primer was able to distinguish between *S*. *alata* and *S*. *tora* by HRM, resulting in great amplification and different melting curve characteristics both for fresh and for dried samples (Fig 2C and 2D). As depicted in Fig 2C and 2D, a significant discrepancy was observed regarding the melting temperature (T_m_) of each species, in that T_m_ of *S*. *alata* was higher than that of *S*. *tora* by 1.07°C (Fig 2C). Moreover, the HRM difference plot displayed a clearly distinctive curve between them because of the shift of *S*. *alata*’s curve from a reference line as *S*. *tora* (Fig 2D). In addition, Bar-HRM using ITS2 enabled determining the admixture of *S*. *alata* and *S*. *tora* corresponding to a dose-dependent manner of contamination (10, 25, 50, 75, and 90% of *S*. *alata* in *S*. *tora*). The admixture between them can be detected at the lowest concentration of 10% (Fig 2E).

### Phytochemicals and antioxidative activity of *S*. *alata* and *S*. *tora*

**Fig 3:**
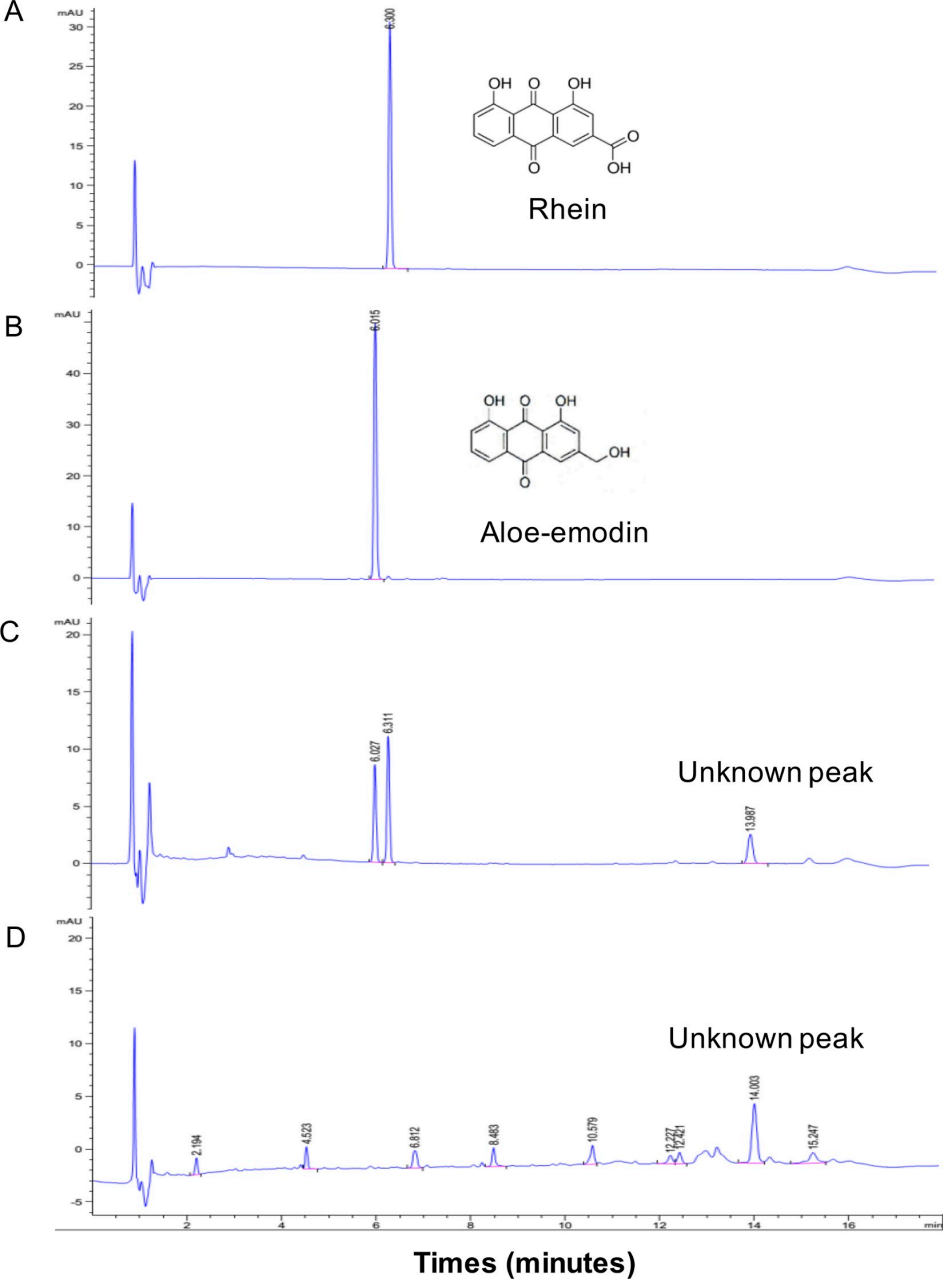
HPLC profiles of the anthraquinone compounds. Rhein (A) and aloe-emodin (B), in the ethanolic extracts of *S*. *alata* (C) and *S*. *tora* (D).

The *S*. *alata* extract was found to contain approximately twice the amount of anthraquinones in the *S*. *tora* extract (Table 1). The *S*. *tora* extract did not contain the anthraquinone derivatives rhein and aloe-emodin (Table 1 and Fig 3), although it had a higher amount of total phenolic compounds and showed a remarkably higher radical scavenging activity than the *S*. *alata* extract (Table 1). Ascorbic acid which was used as a positive antioxidant showed an IC_50_ value of radical scavenging activity at 46.94 ± 2.72 μg/ml. This results indicated that both *Senna* extracts were greater antioxidant than ascorbic acid.

**Table 1:** Total contents of anthraquinones, rhein, aloe-emodin, phenolic compounds, and antioxidation of *S*. *alata* and *S*. *tora*.

Phytochemicals	*S*. *alata*	*S*. *tora*
Total anthraquinones (μg/ml of crude extracts)	26.45 ± 0.84	10.90 ± 0.14
Rhein (μg/ml)	3.65	ND
Aloe-emodin (μg/ml)	1.78	ND
Total phenolic compound (μg gallic acid equivalent/mg of crude extracts)	20.82 ± 0.84	31.08 ± 0.07
Radical scavenging activity IC_50_ of DPPH (μg/ml)	114.38 ± 4.14	16.10 ± 1.76

### Effect of *Senna* extracts and anthraquinone derivatives on preventing cartilage degradation in cartilage explant culture

**Fig 4:**
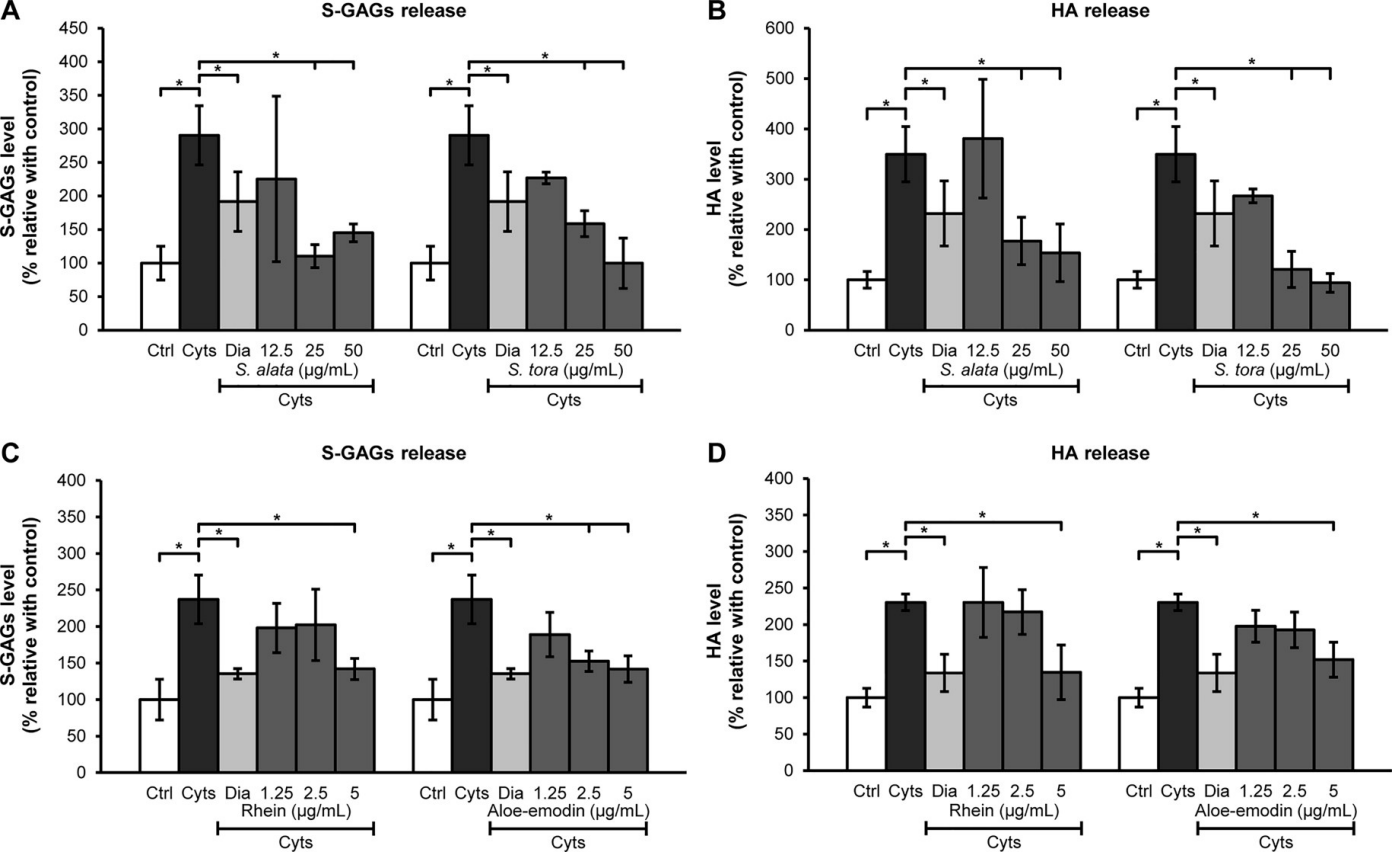
Levels of S-GAGs and HA in the supernatants of the short-term porcine cartilage explant culture. Results are expressed as means ± SD of the proportion between the measured level in each experimental group and the untreated control in triplicate experiments. The asterisk (*) indicates a significant difference at *p* < 0.05. Cyts: the combined cytokines (2ng/mL IL-1β and 4 ng/mL IL-17A); Ctrl: control (untreated group).

In short-term explant culture, the cytokines-induced cartilage degradation considerably increased the release of both sulfated glycosaminoglycans (S-GAGs) and hyaluronic acid (HA) (Fig 4). The cotreatments of the cytokines-induced explants with *S*. *tora* extract at 25 μg/mL led to a significant reduction of S-GAGs and HA in the media, whereas *S*. *alata* extract at 25μg/mL led to a significant decrease of only S-GAGs levels but not HA (Fig 4A and 4B). Rhein and aloe-emodin at 5 μg/mL significantly suppressed the release of both S-GAGs and HA in the culture media (Fig 4C and 4D).

**Fig 5:**
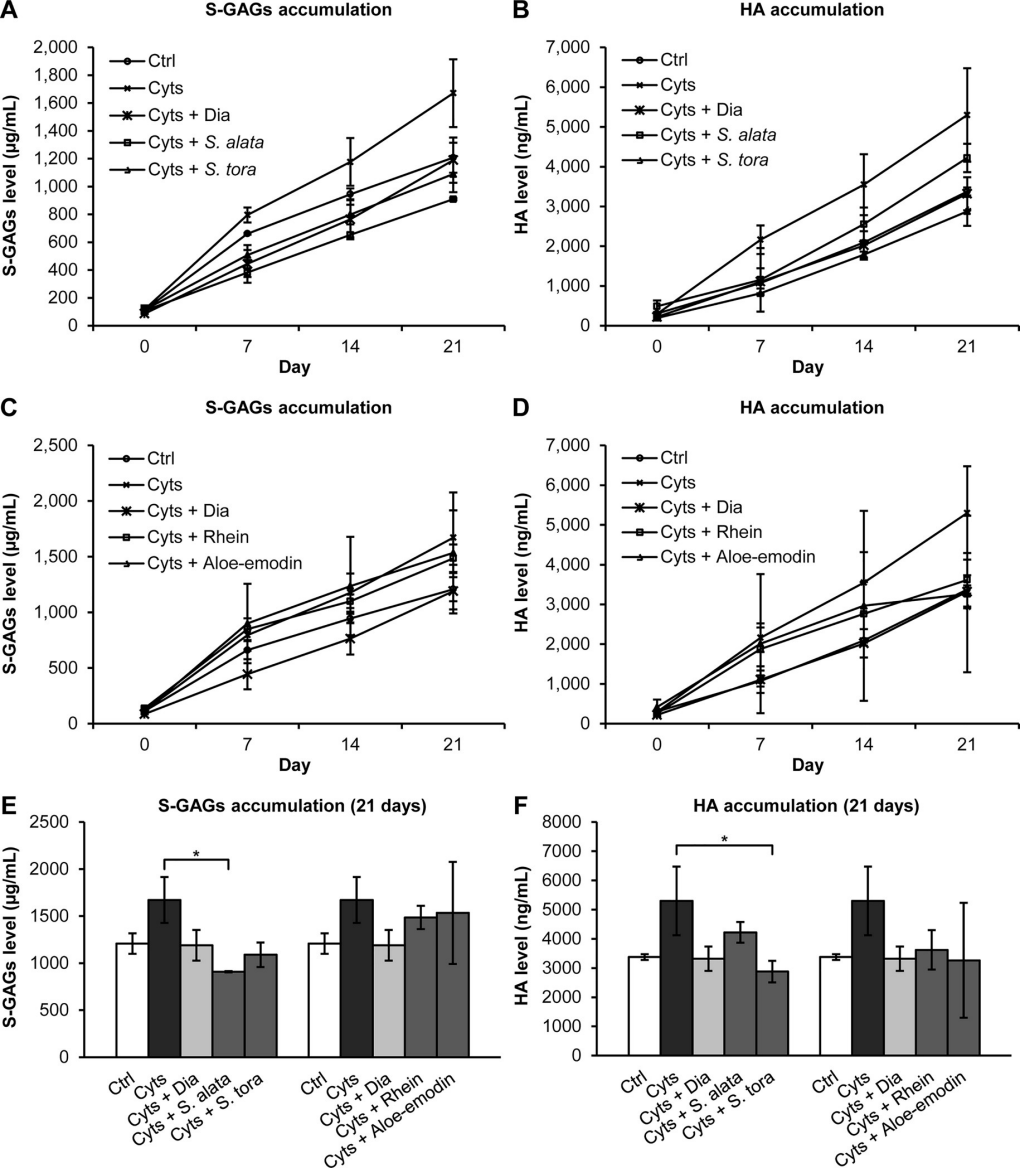
Cumulative release of S-GAGs and HA in the media of the long-term cartilage explant culture. Accumulative values were determined through the sum of the measured S-GAGs or HA levels at each time point and at the previous time point (A–D). Graphs E and F represent the total release of S-GAGs and HA from 21 days of the long-term treatments, respectively. The results are expressed as mean ± SD of triplicate experiments. The asterisk (*) indicates a significant difference at *p* < 0.05. Cyts: the combined cytokines (2 ng/mL IL-1β and 4 ng/mL IL-17A); Ctrl: control (untreated group).

In a long-term explant culture (Fig 5), both *Senna* extracts at a 25 μg/mL concentration led to a great reduction of S-GAGs release from day 7 to day 21, where a significant difference was found in its accumulation, compared to the cytokines-treated group (Fig 5A and 5E). Moreover, both *Senna* extracts tended to reduce HA accumulation in the media throughout the experiment (Fig 5B), although the accumulating value by the 21^st^ day of treatment with only *S*. *tora* extract showed a significant difference in relation to the cytokines-treated group (Fig 5F). Although rhein and aloe-emodin tended to decrease the release of S-GAGs and HA into the culture media, this reduction did not differ significantly from the cytokines-treated group (Fig 5C–5F).

### Histological examination

**Fig 6:**
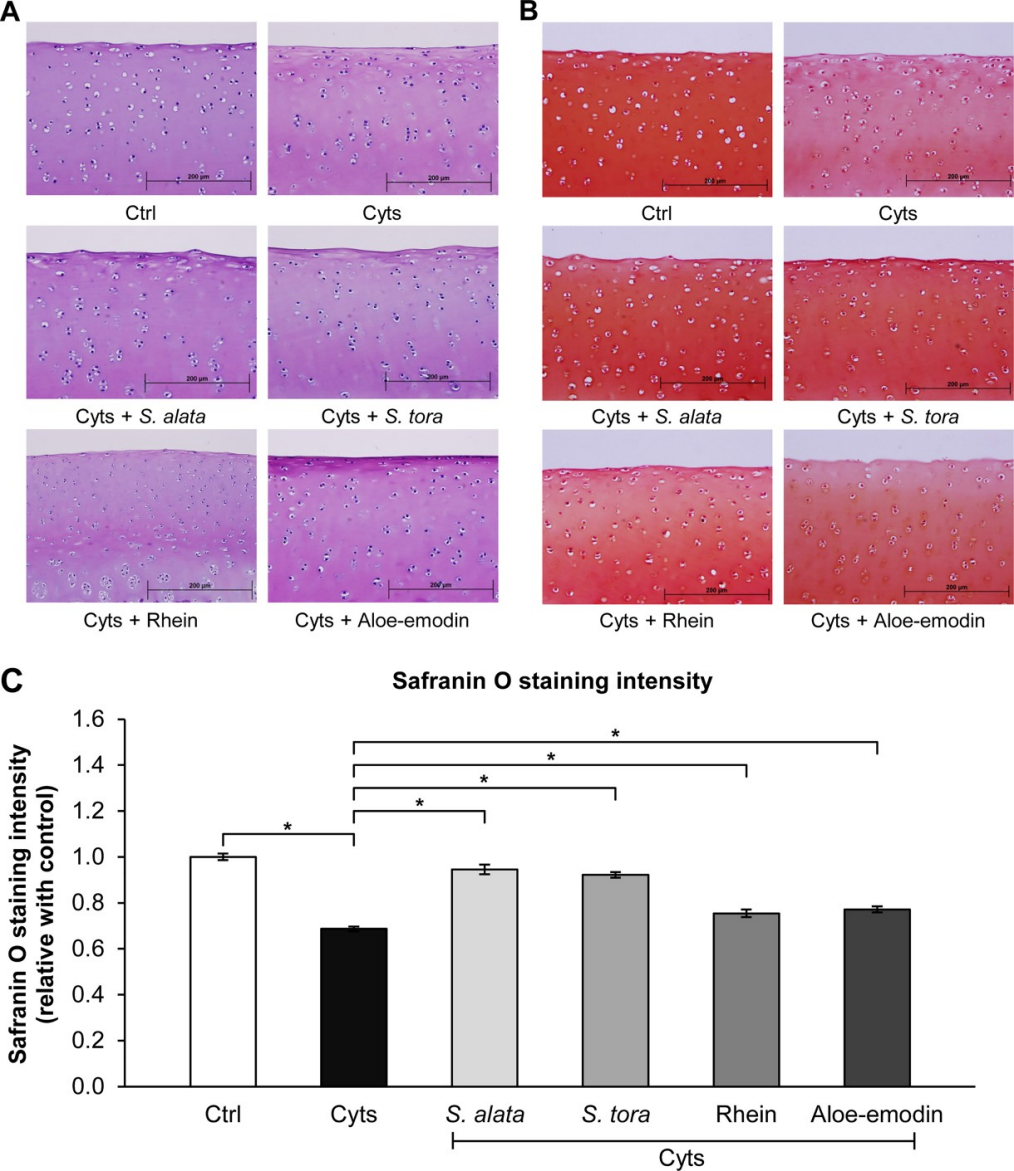
Histological examinations of the cartilage explants after 21 days of the long-term culture. Explants stained with H&E (A) or safranin O (B). Bar graphs (C) represent the color intensity of safranin O staining of cartilage explants. Results are expressed as mean ± SD from triplicate experiments. The asterisk (*) indicates a significant difference at *p* < 0.05. Cyts: the combined cytokines (2 ng/mL IL-1β and 4 ng/mL IL-17A); Ctrl: control (untreated group).

After 21 days, the explant discs were stained with hematoxylin-eosin (H&E) and safranin O to observe the chondrocyte morphology and the proteoglycan amount in the tissue. We noted that the chondrocytes in the superficial tangential and middle transitional zones of all treatments had a normal phenotype and large oval nuclei and contained a ground substance similar to that of the control group (Fig 6A). The nontreated cartilage explant control group, stained with safranin O, presented an intense red color all over the tissue, indicating a high content of cartilaginous proteoglycans (Fig 6B). The presence of a pale red stain during the treatment with combined cytokines indicated a decrease in the proteoglycan content, which appeared to be restored when cotreated with *S*. *alata* or *S*. *tora* extract (Fig 6B and 6C). Regarding the anthraquinone derivatives, although both explants treated with rhein and aloe-emodin seemed to present a highly intense red color when compared to the cytokine-treated group, they exerted a significantly lower activity in preserving proteoglycan depletion than that of both *Senna* extracts, especially aloe-emodin, which was pale red at the superficial zone (Fig 6B).

### Lactate dehydrogenase (LDH) and 3-(4,5-Dimethylthiazol-2-yl)-2,5-diphenyltetrazolium bromide (MTT) assays

**Fig 7:**
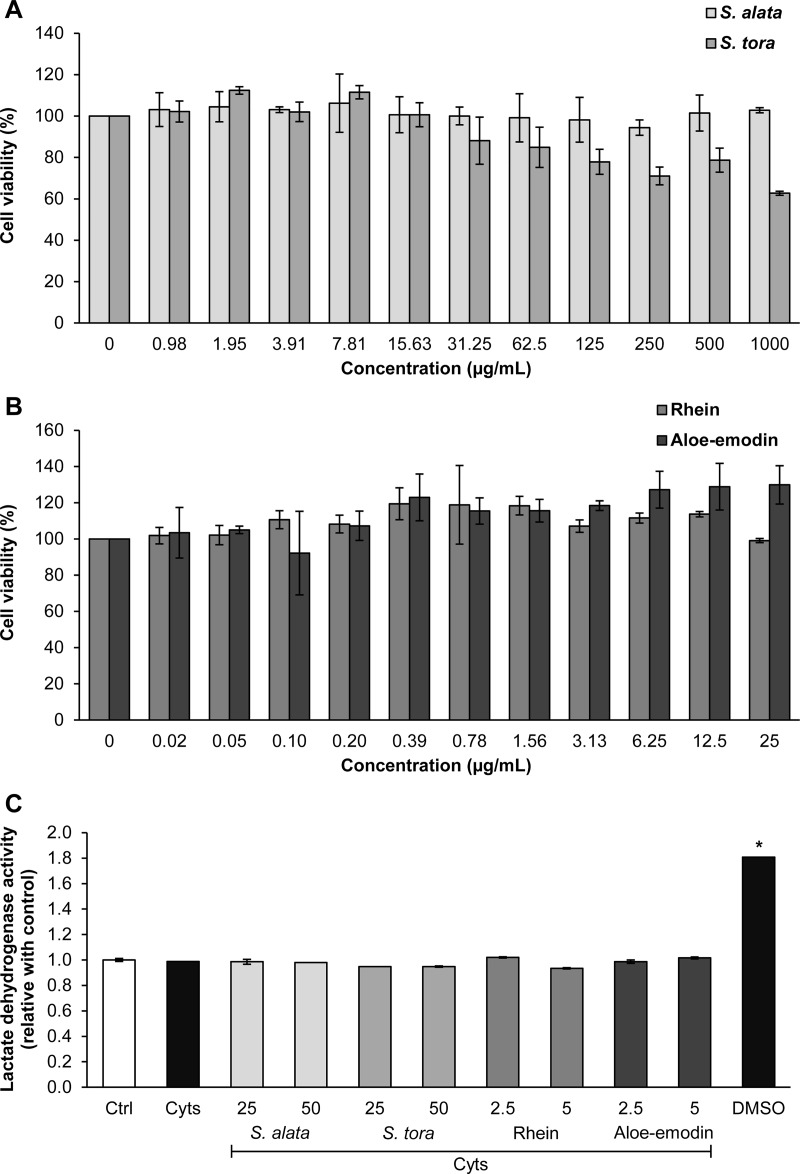
Effect of *Senna* crude extracts, rhein, and aloe-emodin on cell viability of primary porcine articular chondrocytes and cartilage explants. *Senna* crude extracts of MTT assays (A), rhein and aloe-emodin (B), in a primary porcine articular chondrocyte culture for 24 h and LDH assay (C) for seven days of the cartilage explant culture. The culture with 10% DMSO was used as the positive control. Results are presented as mean ± SD of the proportion between the treatment group and the untreated control (Ctrl) in triplicate experiments. The difference between the treatments and untreated control was performed by a Student's t-Test and the asterisk (*) indicates a significant difference at *p* < 0.05. Cyts: the combined cytokines (2ng/mL IL-1β and 4 ng/mL IL-17A).

The MTT assay showed that *S*. *tora* presented an IC_20_ value of <125 μg/mL, whereas neither *S*. *alata* (0–1,000 μg/mL) nor rhein or aloe-emodin (0–25 μg/mL) exerted a cytotoxic effect on porcine articular chondrocyte cultures (Fig 7A and 7B). No significant difference was found regarding LDH activity in the culture media between cartilage explants and the controls (Fig 7C). However, LDH activity was significantly increased in the positive control group, which was treated by 10% dimethyl sulfoxide (DMSO).

## Discussion

### Bar-HRM assay using ITS2 for species differentiation

Misidentification caused by shared morphological characters and/or common name is a serious concern in pharmacology, as it may lead to undesirable effects on consumers and reduced pharmacological properties of medicinal plant ingredients. Among *Senna* species, anthraquinone levels vary, for which excessive consumption may lead to poisoning [41–43]. In order to prevent such scenarios, taxonomic evaluation must be performed based on plant morphology for species identification. In fact, in the case of dried and ground raw materials of medical plants, morphological observations for species identification has proven difficult or even impossible [25–28]. Bar-HRM can be used as an alternative aid for taxonomic evaluation.

Our results show that Bar-HRM using ITS2 allows differentiation between *S*. *alata* and *S*. *tora*. The ITS2 sequence between primers showed a number of different nucleotides among the species. This nucleotide divergence resulted in a shifted melting temperature (T_m_) value between *S*. *alata* and *S*. *tora* of 1.07°C. In addition, the ITS2 marker could determine the admixture of *S*. *alata* and *S*. *tora* according to the contamination ratio (*S*. *alata*:*S*. *tora* as 10, 25, 50, 75, and 90%). PCR products derived from Bar-HRM were sequenced, and as a result, the melting temperature corresponded with the sequences of *S*. *alata* or *S*. *tora* in GenBank with >99%, demonstrating the specificity of Bar-HRM. Here we used only two *Senna* species in our evaluation. However, the specificity test should ideally be done using additional *Senna* species. Our results agree with a recent study by Mishra et al. (2008) [28] that reported the potential of ITS for authentication and detection of *Senna* species. Moreover, it has been suggested that the trnH-psbA spacer region, used as a barcode marker, can produce different size PCR products across different *Senna* species, including *S*. *alata* and *S*. *tora* [28, 29]. Using the trnH-psbA region as a specific marker for species identification of *Senna* species could allow for a candidate barcode marker that could be used in future work.

### Chondroprotective properties of *S*. *alata* and *S*. *tora*

In the present study, *Senna* extracts (at a 25 μg/mL concentration) revealed a potent chondroprotective activity against cytokine-induced cartilage degradation both in short- and in long-term cultures of porcine cartilage explants. This activity was evaluated by the reduction of S-GAGs and HA release, which was consistent with the increase of accumulated proteoglycans in the cartilage tissue, as clearly demonstrated by Safranin O staining. Interestingly, our findings suggest that the anthraquinone derivatives, which are generally found in *Senna*, rhein and aloe-emodin, do not constitute the main bioactive compounds of *Senna* extracts that are responsible for the chondroprotective properties, as these were found in the extract of *S*. *tora* but not in that of *S*. *alata*.

With regard to the role of proinflammatory cytokines in cartilage destruction, IL-1β triggers cartilage degradation through the induction of inflammatory mediators (NO and PGE2) and matrix-degrading enzymes [1, 2]. IL-17A, a T-cell-derived cytokine, not only promotes proteoglycan loss, but also was recently found to induce collagen breakdown from cartilage [6, 44]. In the present study, explants treated with a combination of IL-1β and IL-17A markedly stimulated the release of the cartilage biomolecules S-GAGs and HA into the media, as well as the loss of proteoglycan from the explant tissues. These results indicate a promoting effect of these cytokines on matrix turnover and cartilage destruction, which mimics the OA condition.

It has been reported that the methanolic extract of *S*. *tora* leaves exerts an anti-inflammatory effect [14], down-regulating the expression of inducible nitric oxide synthase (iNOS), cyclooxygenase-2 (COX-2), and matrix metalloproteinases 2 and 9 via the reduction of the NF-κB pathway [45]. Hexane extracts of *S*. *alata* leaves have been found to protect cartilage from degradation in a rat model of complete Freund’s adjuvant-induced arthritis [24]. In our study, both *S*. *tora* and *S*. *alata* were extracted using ethanol. It is, therefore, possible that the active compounds of *S*. *alata* ethanolic extract, which is responsible for the chondroprotective effect evaluated in the present study, are included in the same groups of active components found in the hexane extract of *S*. *alata* leaves.

The chondroprotective effects of the anthraquinone compounds rhein and aloe-emodin present in the *Senna* genus, excluding *S*. *tora*, were evaluated. It was noted that both rhein and aloe-emodin, at a concentration of <2.5 μg/mL, could not reduce the release of S-GAGs and HA, even though the weak preservation of the proteoglycan content by these anthraquinone compounds was observed in the long-term cartilage explant culture. This was unexpected given that several previous studies claim that rhein is an effective inhibitor of proteoglycan degradation, contributing to the maintenance of cartilage integrity [19–21]. Aloe-emodin is an effective inducer of chondrogenic differentiation via enhancement of the expression of chondrogenic markers such as collagen II, collagen X, BSP, and RunX2 [24]. It can inhibit lipopolysaccharide-induced inflammation responses in RAW264.7 macrophages via NF-κB, JNK, ERK, p38, and Akt pathways [46].

Both rhein and aloe-emodin exert a beneficial effect on cartilage integrity through both anti-degradation and matrix synthesis. However, the ability to protect against cytokine-induced cartilage damage was found to be weak, which may be due to two reasons. (i) Model generation may be a crucial factor behind such contradictory results since, in this study, we used a combination of the cytokines IL-1β and IL-17A to induce cartilage degradation, while in most previous studies, either IL-1α- or IL-1β-induced porcine cartilage explants were used [19–21]. The mechanisms/pathways triggering cartilage destruction may differ for inductions with different stimuli. It has previously been reported that IL-1β- or IL-17A-induced OA chondrocytes displayed increased collagenase-3 production though distinct complexes of activating protein-1 (AP-1) [44]. However, the mechanisms of IL-17A in combination with IL-1β and other cytokines in chondrocytes is not fully understood. Confirming the mechanism behind the effect of rhein in models induced by different stimuli, in particular the condition of combined inflammatory cytokines, should be the subject of future investigations. (ii) Concentrations of rhein and aloe-emodin used in this study could be insufficient to inhibit cartilage destruction induced by combined IL-17A and IL-1β. Typically, the required dose of rhein to effectively inhibit cartilage destruction in IL-1α- or IL-1β-treated explants or chondrocytes is above 10 μM (or 2.84 μg/mL) [19–21]. On the other hand, the required dose of aloe-emodin for effective chondrogenic differentiation is above 10μM (or 2.70 μg/mL) [24, 46]. In the present study, the tested concentrations of rhein and aloe-emodin were 1.25–5 μg/mL. Therefore, we believe that the concentration of rhein or aloe-emodin necessary to effectively treat combined cytokine-stimulated explants may be higher than that required to treat single cytokine-stimulated explants.

Based on our results, rhein and aloe-emodin present in *Senna* extracts may not be the major active compounds preventing cartilage destruction for two main reasons. (i) Phytochemical analysis revealed that *S*. *tora* extract has a small amount of total anthraquinones, among which rhein and aloe-emodin were undetectable. Nevertheless, this extract showed superior activity for maintenance of cartilage integrity, similar to the effect of *S*. *alata* extract, in which rhein (3.65 μg/mg of crude extract) and aloe-emodin (1.78 μg/mg of crude extract) were present. (ii) Although the *S*. *alata* extract at 50 μg/mL contained a small amount of rhein (0.178 μg/mL) and aloe-emodin (0.089 μg/mL), its anti-cartilage degradation effect seemed to be greater than the effect of pure rhein (5 μg/mL; 28-fold versus *S*. *alata* at 50 μg/mL) and aloe-emodin (5 μg/mL; 56-fold versus *S*. *alata* at 50 μg/mL). Moreover, *S*. *tora* extract had a higher amount of total phenolic compounds and unknown anthraquinone derivatives and presented a significantly higher antioxidant activity than *S*. *alata*. We speculate that the potent chondroprotection of the ethanolic extract of *S*. *tora* and *S*. *alata* may result from an synergistic/additive effect of some other anthraquinones, such as emodin and chrysophanol [17] or from other phytoconstituents (phenolic compounds).

## Conclusion

Taken together, we report several significant findings. (i) Presently, commercial *Senna* products distributed on the market are available in various forms, including powder, dried, ground leaves, or stem cuttings, which can lead to difficult species delimitation. Bar-HRM using ITS2 is required to ensure the quality of herbal medicinal plant products for consumers’ health. Our results show that Bar-HRM using ITS2 serves as a promising tool, given that it is a reliable, highly sensitive, economic, and a rapid method to authenticate *S*. *alata* and *S*. *tora* from their raw materials and even in commercial products. (ii) Both ethanolic extracts of *S*. *alata* and *S*. *tora* possess a anti-cartilage destruction from the cytokine (IL-1β- and IL-17A) induction by halting matrix degradation such as proteoglycan although the absence of rhein and aloe-emodin was seen in *S*. *tora*. This evidence indicated that the anthraquinones in *Senna* might not be a major bioactive compound responsible for chondroprotection in porcine cartilage.

## Supporting information

S1 File Data availability for Chondroprotection of *Senna species*. *All raw data in* the file was used for generating Figs 3–6.
(XLSX)

## Abbreviations

S-GAGs: Sulfated glycosaminoglycans
HA: Hyaluronic acid
ITS2: Internal transcribed sequence 2
Bar-HRM: DNA barcoding coupled with high resolution melting analysis
